# Out-of-pocket medical costs in relation to resection of colorectal liver metastases in the Australian healthcare system

**DOI:** 10.1007/s00520-025-09669-2

**Published:** 2025-06-24

**Authors:** Nazim Bhimani, Rebecca Seton, David Chan, Mbathio Dieng, Patrick J. Kelly, Thomas J. Hugh

**Affiliations:** 1 Upper Gastrointestinal Surgical Unit, Clinical Administration 8 A, Acute Services Building, Royal North Shore Hospital, St Leonards, NSW 2065 Australia; 2 Faculty of Medicine and Health, University of Sydney, Sydney, NSW Australia; 3 Colorectal Surgical Department, Royal North Shore Hospital, St Leonards, NSW Australia; 4 Medical Oncology Department, Royal North Shore Hospital, St Leonards, NSW Australia; 5 GenesisCare, North Shore Health Hub, St Leonards, NSW Australia; 6 Sydney School of Public Health, University of Sydney, Sydney, NSW Australia; 7 Northern Clinical School, University of Sydney, Sydney, NSW Australia

**Keywords:** Colorectal liver metastases, Out-of-pocket costs, Financial burden, Financial stress

## Abstract

**Purpose:**

The cost of treating colorectal liver metastases (CRLM) places a financial burden on the healthcare system; however, there is limited research on the out-of-pocket (OOP) costs to patients. This study aimed to assess the direct medical OOP costs on patients who undergo liver resection for CRLM and evaluate their financial worry, stress, and difficulty.

**Methods:**

This was a retrospective cohort analysis of the OOP costs for patients who underwent potentially curative resection of CRLM in Sydney, Australia, between 2010 and June 2021. These costs were determined in Australian dollars from the diagnosis of liver metastases to their last follow-up. Patients completed a series of questions relating to financial worry, stress, and difficulty.

**Results:**

In total, 121 patients underwent liver resection, of which 85 were alive at a median follow-up of 5.3 years (1.8–13.7). There was a 59% response rate (50/85). Specialist consultation costs varied, with a median OOP cost of $393.35. Liver surgical treatment incurred the highest median OOP cost of $1011.29 (range $0–$7246.54). There were minimal OOP costs for chemotherapy and no OOP costs for radiation oncology. Most patients were not worried about the OOP costs (60%) and said there was no impact on their ability to make ends meet (64%) or had no effect on their finances (56%).

**Conclusion:**

This study demonstrates that patients who undergo liver resection for CRLM and have long-term survival have moderate OOP costs. Most patients were not worried or stressed with the amount they had to pay OOP.

**Supplementary Information:**

The online version contains supplementary material available at 10.1007/s00520-025-09669-2.

## Introduction

The cost of treating cancer can place a heavy burden on the healthcare system, patients, and their families [1]. From diagnosis to treatment, and then from survivorship to end-of-life care, the costs for some patients may be significant. These can be divided into medical and non-medical costs [2]. Medical costs include consultations, investigations, hospital admissions, surgical or radiological interventions, and medications [2, 3]. Non-medical costs include transport, food, and loss of productivity [2, 3], and these later are usually the sole responsibility of the patients and their caregivers.

The Australian healthcare system is complex, and there is inconsistent integration of health service delivery, which consists of both public and private entities. Public healthcare includes medication support provided by the Pharmaceutical Benefits Scheme (PBS), medical services support through the Medicare Benefits Scheme (MBS), and public hospital services funded by state governments [4]. Some health services for treating cancer are also provided by private hospitals, private clinics, and private organisations, and there is partial funding support for these from private health insurance. Regardless, patients often still incur additional out-of-pocket (OOP) costs, as the fees charged by private health service providers vary by the type of provider and their location.

Despite having private insurance cover, patients treated in private hospitals may face higher OOP costs for surgical interventions and procedures compared to public hospital patients due to the gaps in coverage. These gaps may include paying the hospital excess fees or differences between scheduled rebates and actual costs charged by specialists. Despite most people knowing and accepting that there will likely be OOP costs, 44.8% of Australians elect to take out private insurance, and in New South Wales, this rate is even higher at 46.2% [5]. Reasons for selecting private treatment over public treatment vary but include a sense of certainty about timely treatment, the ability to choose a specialist, and a desire to avoid the government imposed surcharge for not having private health insurance [6].

Navigating through a fragmented healthcare system can be difficult for patients and their families, with the need to access care from different healthcare providers in different locations. This is problematic given the disease burden in the community, with 50% of Australians having at least one chronic condition requiring integrated care [6].

In Australia, colorectal cancer (CRC) is the third most common cancer [7]. Unfortunately, many patients either present with metastatic disease or go on to develop metastatic disease [8]. Those with isolated colorectal liver metastases (CRLM) may be able to undergo curative liver resection, with an expectation of a cure and a return to normal life [9]. In many countries, CRC is also associated with significant costs for both the patient and society. Internationally, the average overall cost of treating metastatic CRC ranges from $12,346 to $293,461 [10]. To date, there has been no research focusing on the OOP medical costs and the associated financial stress for patients treated for CRLM.

Given the multifaceted Australian healthcare system, we hypothesise that the median OOP medical costs will be variable, but that patients who achieve long-term cure are not likely to express financial burden or worry about these costs. This study aimed to identify the median OOP medical costs in patients who underwent a potentially curative liver resection of CRLM in the Australian health system and to examine any financial burden or worry about treatment costs.

## Methods

A single-unit cohort study of patients who had potentially curative resection for CRLM between 2010 and June 2021 at two hospitals (Royal North Shore Hospital and North Shore Private Hospital) was undertaken. Ethics approval was given by the Northern Sydney Local Health District Human Research Ethics Committee (LNR/11/HAWKE/258). Patients with CRLM who underwent curative resection and had at least 1-year follow-up were retrospectively identified from a prospectively collected database. Patients who underwent resection before 2010 were not included in the study as biological agents (cetuximab and bevacizumab) were not available or were not routinely used before this.

The questionnaire was developed using validated financial burden questions from studies by Markman et al. and Veenstra et al. [11, 12]. A list of these questions can be found in the Appendix. Patients were contacted by telephone before posting a questionnaire. Patients who did not return the questionnaire after 3 months were contacted again and encouraged to participate. The questionnaire consisted of demographic details and financial burden and worry. Demographic details included gender, date of birth, marital status, employment status, education, household income, private health insurance, and the number of adults and children in the house. Financial burden and worry included questions on how worried patients were about their financial problems measured on a five-point Likert scale, the financial burden on the ability to make ends meet, and the impact it had on finances on a seven-point Likert scale.

A detailed patient timeline from diagnosis of liver metastases to 2022 was constructed, listing all public and private hospital admissions, private consultation visits, operation details, chemotherapy, and radiation therapy doses. Costs of imaging, blood tests, and general practitioner visits were not examined as it was assumed that Medicare (funded by the government) covered these costs and that there were mostly no OOP costs. Surgical and procedural costs only included the primary surgeon fees, not the assisting surgeon or anaesthetist’s fees. Due to the difficulty of determining what medications were taken and how much, this was also not considered for this study. For the sake of a consistent approach and because this was relatively easy to measure, the costs for consultations and operations were assumed to be the Australian Medical Association (AMA) fee, with the OOP cost calculated as the difference between the AMA fee and the Medicare scheduled rebate. Radiation therapy, chemotherapy, immunotherapy, and radiation oncology and medical oncology consultations performed in the public hospital incurred no OOP costs. For the same treatment obtained in private hospitals, patients only paid the yearly hospital excess to the private health fund and paid the OOP cost set by the PBS for chemotherapy. All costs were adjusted for inflation using an inflation calculator (https://www.rba.gov.au/calculator/) and presented in Australian dollars as of 2022.

Information obtained from the database included demographical data (age, sex, marital status, employment status, education level, household income), pre-existing medical conditions (Charlson comorbidity index (CCI) and American Society of Anesthesiologists (ASA)), radiological investigations (timing, primary side), neoadjuvant treatments (chemotherapy ± biological agents, radiotherapy, transarterial chemoembolisation (TACE), selective internal radiation therapy (SIRT), live ablation, portal vein embolisation), intra-operative details (operative technique, extent of liver resection, resection margin, tumour differentiation), post-operative outcomes (complications, length of stay, recurrence), and post-operative treatment (chemotherapy ± biological agents, liver ablation, locoregional therapy). Neoadjuvant chemotherapy or biological agent treatments were defined as treatment given at any time 6 months before the liver resection. The extent of the liver resection was defined as either major or minor, where a major liver resection was a resection of at least three contiguous Couinaud segments. Long-term survival was defined as greater than or equal to 5 years.

Categorical variables were presented as counts and percentages and analysed using Chi-squared tests or Fisher’s exact test. Continuous variables were expressed as a mean and standard deviation for normally distributed data and analysed using *T* tests. Continuous variables not normally distributed were expressed as a median and range and analysed using the Mann–Whitney *U* test. All statistical analyses were performed using Stata® BE for Windows® version 17.1 (StataCorp, College Station, TX, USA).

## Results

**Fig. 1:**
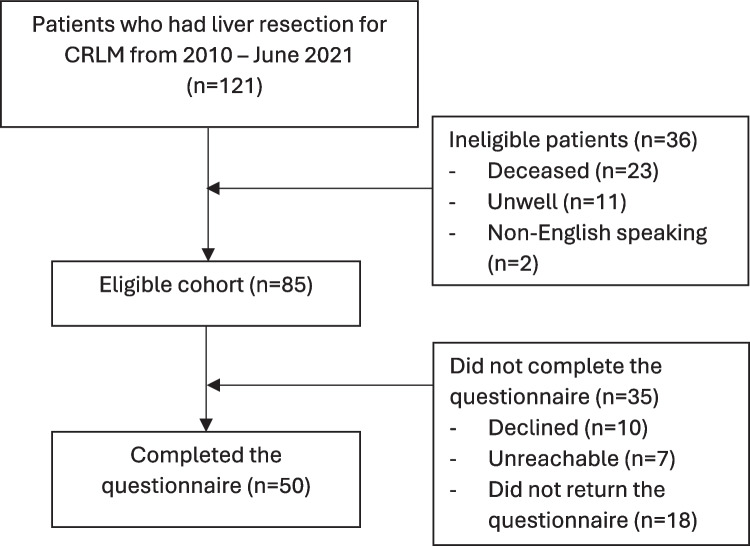
Flow chart of patients included in the study

From 2010 to June 2021, 121 patients underwent liver resection for CRLM. Of these, 23 patients (19%) were deceased, 11 patients (9%) were too unwell to complete the questionnaire, and two patients (2%) were deemed unsuitable for the study as they were unable to speak English. The cohort of interest consisted of 85 patients. Ten patients declined to complete the questionnaire for an unspecified reason, seven patients were unreachable after three attempts at contact, and 18 patients did not return the questionnaire and were therefore excluded. The final cohort included 50 respondents (59% response rate), and these are the subjects of this study, as shown in Fig. 1. The median follow-up from diagnosis of CRLM to determine OOP costs was 5.3 years (range 1.8–13.7).

Demographic characteristics are shown in Table 1. Most survey participants were men (60%), and the median age at the time of completion of the survey was 69 years. Most were either married or living as a married couple (72%), were retired (60%), and had at least completed high school (84%). When the patient was the primary earner in the household, 38% were earning ≥ $80,000 per year. In the study cohort, 66% of patients had private health insurance.

**Table 1:** Demographic characteristics

Variable	
Gender, *n* (%)	
Male Female	30 (60) 20 (40)
Median age when survey was completed (range)	69 (33–85)
ASA, *n* (%)	
1 2 3	32 (64) 16 (32) 2 (4)
Median Charlson comorbidity index (range)	8 (6–12)
Marital Status, *n* (%)	
Single Married or living as a married couple Separated Divorced Widowed	4 (8) 36 (72) 3 (6) 3 (6) 4 (8)
Employment status, *n* (%)	
Full-time work Part-time work Seeking work Retired	13 (26) 6 (12) 1 (2) 30 (60)
Education level, *n* (%)	
Primary Some high school Completed high school Vocational education training Undergraduate University Postgraduate University	1 (2) 7 (14) 15 (30) 12 (24) 12 (24) 3 (6)
Annual household income, *n* (%)	
Less than $19,999 $20,000 to $39,999 $40,000 to $59,999 $60,000 to $79,999 $80,000 to $99,999 $100,000 to $119,999 $120,000 to $139,999 $140,000 to $159,999 $160,000 to $179,999 Above $200,000	5 (10) 11 (22) 6 (12) 9 (18) 3 (6) 6 (12) 1 (2) 2 (4) 2 (4) 5 (10)
Main earner in the household, *n* (%)	
Patient with cancer Spouse Shared between survivor/spouse	22 (44) 10 (20) 18 (36)
Private health Insurance, *n* (%)	
Yes No	33 (66) 17 (34)
Adults in household, *n* (%)	
1 2 3 or more	9 (18) 32 (64) 9 (18)
Children in household, *n* (%)	
0 1 or 2	39 (78) 11 (22)

Table 2 describes the tumour characteristics and treatment received. Most patients had the primary tumour removed first (82%), had a Dukes C tumour (62%), had a left-sided primary tumour (82%), presented with synchronous disease (56%), received neoadjuvant chemotherapy (82%), had a minor liver resection (76%), did not have a complication after liver resection (59%), received postoperative chemotherapy (96%), and did not develop recurrent disease (58%).

**Table 2:** Tumour biology and treatment received

Variable	*n* (%)
Primary resection details	
Order of resection	
Primary first Liver first Combined	41 (82) 2 (4) 7 (14)
Dukes stage	
Dukes A Dukes B Dukes C	3 (6) 16 (32) 31 (62)
Primary site	
Colon Rectum	30 (60) 20 (40)
Sidedness	
Left Right	41 (82) 9 (18)
Timing of liver metastases	
Metachronous Synchronous	22 (44) 28 (56)
Treatment prior to liver resection	
Neoadjuvant chemotherapy	41 (82)
SBRT OR chemoradiotherapy	4 (8)
TACE	0 (0)
SIRT	0 (0)
RFA or MVA	0 (0)
Portal vein embolisation	1 (2)
None	5 (10)
Liver operative details (***n*** = 59)	
Operative technique	
Open Laparoscopic Laparoscopic converted to open	51 (87) 6 (10) 2 (3)
Liver resection	
Major Minor	14 (24) 45 (76)
Median length of stay in days (range)	7 (3–30)
Complications	24 (41)
Tumour differentiation	
Well Moderate Poor Not stated	5 (9) 29 (51) 1 (2) 22 (39)
Treatment and outcomes following liver resection(s) (n = 50)	
Chemotherapy	48 (96)
SBRT	8 (16)
Chemoembolisation/TACE	1 (2)
SIRT	1 (2)
ETOH ablation	0 (0)
RFA or MVA	2 (4)
Recurrence	21 (42)

Table 3 summarises the median direct medical OOP costs for treating CRLM. The OOP cost of a consultation with a colorectal surgeon, liver surgeon, and gastroenterologist were similar. The complexity of liver resection was reflected in the higher OOP costs with a median OOP cost of $1011.29 (range 0–7246.54), compared to primary colorectal surgery with a median cost of $0 (range 0–4632.25). Another likely reason for this difference is that costs were determined from the diagnosis of liver metastases rather than the diagnosis of the primary bowel cancer, and so some costs associated with the treatment by the colorectal surgeon were not included in the analysis. There are no costs for consultations with a radiation oncologist or for radiation therapy, as all patients were treated in public hospitals and consequently would not have had an OOP cost. Similarly, the median cost for chemotherapy was only $29 (range 0–57502) as patients who were treated in the public hospital did not incur an OOP fee, and patients who had treatment in the private hospital only paid the yearly hospital excess to the private health fund and paid the OOP cost set by the PBS. One patient who self-funded their chemotherapy and immunotherapy treatment incurred a cost of approximately $57,500 for their treatment. The overall median OOP cost per person is $4091.88, equating to $673.50 per year. In this study, 15 patients (30%) had their entire treatment in the public sector, 14 patients (28%) had their entire treatment in the private sector, and 21 patients (42%) had a mix of treatment in the public and private sectors.

**Table 3:** Direct medical OOP costs with median and range for treating CRLM in AUD by treatment facility

Treatment	Overall OOP cost (*n* = 50)
Liver	
Consultations Surgical treatment	186.3 (0–363.4) 1011.29 (0–7246.54)
Colorectal	
Consultations Surgical treatment	204.98 (0–1058) 0 (0–4632.25)
Chemotherapy	29 (0–57502)
Radiation oncology	0 (0–0)
Gastroenterologist	
Consultations Procedures	186.7 (0–492.85) 0 (0–174.95)
Hospital excess	750 (0–5000)
Overall costs	4091.88 (232–57,690.45)

Table 4 summarises the financial burden and worry in the diagnosis, treatment, and follow-up of CRLM. Most patients were not worried about OOP costs or were only slightly worried about their financial problems (60%) during or after their treatment. Similarly, most patients suggested that there was no impact on their ability to make ends meet (64%) or that there was no impact on their finances (56%). Sixty per cent of patients used their savings to pay for their OOP while another 20% used savings and other sources, including borrowing money or taking a loan, cutting down on spending on food, clothes, recreational activities or expenses in general, selling stocks or investments. One patient decided to sell their home as part of downsizing but also to help fund their cancer care. Despite some patients having to find additional funds to cover the cost of their treatment, only 22% of patients in this cohort felt that they spent more money than they had expected, while the majority (68%) thought that the amount of medical benefits received was either more than they expected or about what they expected. Only 26% of patients considered the cost when selecting a treatment course. There were no patients who decided not to have a recommended cancer treatment because of the costs.

**Table 4:** Financial burden and worry in the diagnosis, treatment, and follow-up of CRLM

Variable	*n* (%)
Worry about financial problems	
Not at all Slightly Moderately/somewhat Very much Extremely	22 (44) 8 (16) 10 (20) 10 (20) 0 (0)
Impact on the ability to make ends meet	
Very difficult Difficult Somewhat difficult Normal Somewhat easy Easy Very easy	3 (6) 3 (6) 12 (24) 25 (50) 1 (2) 3 (6) 3 (6)
Illness has had no impact on finances	
Strongly disagree Disagree Somewhat disagree Neither agree nor disagree Somewhat agree Agree Strongly agree	4 (8) 10 (20) 8 (16) 5 (10) 12 (24) 6 (12) 5 (10)
How did they pay	
Used savings Borrowed money or took a loan Could not make payments on credit cards or other bills Cut down on spending on food and/or clothes Cut down on spending on health care for other family members Cut down on recreational activities Cut down on expenses in general Sold some personal items/possessions, e.g. car, jewellery Sold stocks or investments Sold their home	43 (86) 3 (6) 1 (2) 5 (10) 0 (0) 5 (10) 16 (32) 1 (2) 3 (6) 1 (2)
Describe your reaction to the cost of treating cancer	
Spent more money than I had expected Spent about what I expected Spent less than I expected	13 (26) 21 (42) 16 (32)
Describe your reaction to how your medical benefits covered the cost of treating cancer	
Contributed more money than I expected Contributed about what I expected Contributed less money than I had expected	12 (24) 22 (44) 16 (32)
When choosing a treatment course for your cancer, did you consider the cost of treatment?	
No, not at all Yes, a little bit Yes, a great deal	37 (74) 11 (22) 2 (4)
Did you decide not to have a recommended cancer treatment because it was too expensive?	
No Yes Do not know/not sure	48 (96) 0 (0) 2 (4)

Tables 5 and 6 examine the relationship between patient and disease factors and financial worry and financial difficulty, respectively. The following factors had no impact on financial worry or financial difficulty: having multiple liver resections, undergoing any chemotherapy, whether they were on active treatment, had recurrent disease, the annual household salary, cohabitation status, education level, who the primary household earner was, having private health insurance, or gender of the patient. Interestingly, patients who had complications following liver resection expressed greater financial difficulty (67% vs. 38%, *p* = 0.048); however, there was no difference in financial worry (*p* = 0.729). Similarly, working patients expressed greater financial difficulty compared with non-working patients (61% vs. 25%, *p* = 0.012); however, there was no difference in financial worry (*p* = 0.153). Patients with children had greater financial worry than those who did not have children (40% vs. 10%, *p* = 0.017); however, there was no difference in financial difficulty (*p* = 0.147). Younger patients were more likely to have financial worry (60% vs. 20%, *p* = 0.004) and financial difficulty (78% vs. 13%, *p* < 0.001) than older patients.

**Table 5:** Relationship between financial worry and patient/disease factors

	Total cohort (*n* = 50)	No or slightly worried (*n* = 30)	Moderate or very much (*n* = 20)	*p* value
Liver resection complications				0.729
Yes No	24 (48) 26 (52)	15 (50) 15 (50)	9 (45) 11 (55)	
Multiple liver resections				0.450
Yes No	8 (16) 42 (84)	6 (20) 24 (80)	2 (10) 18 (90)	
Any chemotherapy				0.118
Yes No	36 (72) 14 (28)	19 (63) 11 (37)	17 (85) 3 (15)	
Currently receiving treatment				0.676
Yes No	11 (22) 39 (78)	6 (20) 24 (80)	5 (25) 15 (75)	
Recurrence				0.349
Yes No	21 (42) 29 (58)	11 (37) 19 (63)	10 (50) 10 (50)	
Annual household income				0.812
< $80,000 ≥ $80,000	31 (62) 19 (38)	19 (63) 11 (37)	12 (60) 8 (40)	
Cohabitation status				0.353
Single Couple	14 (28) 36 (72)	10 (33) 20 (67)	4 (20) 16 (80)	
Employment status				0.153
Employed Non-employed	19 (38) 31 (62)	9 (30) 21 (70)	10 (50) 10 (50)	
Education level				0.487
Tertiary educated No tertiary education	27 (54) 23 (46)	15 (50) 15 (50)	12 (60) 8 (40)	
Children in household				0.017
0 1 or 2	39 (78) 11 (22)	27 (90) 3 (10)	12 (60) 8 (40)	
Main household earner				0.999
Patient with cancer Spouse Shared	22 (44) 10 (20) 18 (36)	13 (43) 6 (20) 11 (37)	9 (45) 4 (20) 7 (35)	
Private health insurance				0.626
Yes No	33 (66) 17 (34)	19 (63) 11 (37)	14 (70) 6 (30)	
Age at survey				0.004
< 65 ≥ 65	18 (36) 32 (64)	6 (20) 24 (80)	12 (60) 8 (40)	
Gender				0.077
Male Female	30 (60) 20 (40)	15 (50) 15 (50)	15 (75) 5 (25)

**Table 6:** Relationship between financial difficulty and patient/disease factors

	Total cohort (*n* = 50)	Difficult (*n* = 18)	Not difficult (*n* = 32)	*p *value
Liver resection complications				0.048
Yes No	24 (48) 26 (52)	12 (67) 6 (50)	12 (38) 20 (62)	
Multiple liver resections				0.999
Yes No	8 (16) 42 (84)	3 (17) 15 (83)	5 (16) 27 (84)	
Any chemotherapy				0.744
Yes No	36 (72) 14 (28)	14 (78) 4 (22)	22 (69) 10 (31)	
Currently receiving treatment				0.147
Yes No	11 (22) 39 (78)	6 (33) 12 (67)	5 (16) 27 (84)	
Recurrence				0.145
Yes No	21 (42) 29 (58)	10 (56) 8 (44)	11 (34) 21 (66)	
Annual household income				0.055
< $80,000 ≥ $80,000	31 (62) 19 (38)	8 (44) 10 (56)	23 (72) 9 (28)	
Cohabitation status				0.999
Single Couple	14 (28) 36 (72)	5 (28) 13 (72)	9 (28) 23 (72)	
Employment status				0.012
Employed Non-employed	19 (38) 31 (62)	11 (61) 7 (39)	8 (25) 24 (75)	
Education level				0.178
Tertiary educated No tertiary education	27 (54) 23 (46)	12 (67) 6 (33)	15 (47) 17 (53)	
Children in household				0.147
0 1 or 2	39 (78) 11 (22)	12 (67) 6 (33)	27 (84) 5 (16)	
Main household earner				0.298
Patient with cancer Spouse Shared	22 (44) 10 (20) 18 (36)	9 (50) 5 (28) 4 (22)	13 (41) 5 (15) 14 (44)	
Private health insurance				0.999
Yes No	33 (66) 17 (34)	12 (67) 6 (33)	21 (66) 11 (34)	
Age at survey				< 0.001
< 65 ≥ 65	18 (36) 32 (64)	14 (78) 4 (22)	4 (13) 28 (87)	
Gender				0.904
Male Female	30 (60) 20 (40)	11 (61) 7 (39)	19 (59) 13 (41)	

## Discussion

The OOP costs for patients who had a potentially curative resection of CRLM in Australia are manageable, and, in general, patients do not often report financial difficulty or stress. These findings differ from the OOP costs for CRC in other parts of the world, such as China, the USA, and Malaysia [13–15]. This is the first Australian study to assess the median OOP cost from the diagnosis of CRLM and the clinical factors associated with financial worry and financial difficulty. OOP costs were highest in relation to the surgical treatment rather than medical or radiation oncological treatments. Patients who reported financial worry and difficulty were mostly younger patients who have children.

Two other international studies of patients with CRC found that OOP costs related to surgical treatment are high compared to radiotherapy and chemotherapy costs [16, 17]. Azzani et al. found similar findings across all cancer stages. Newton et al. looked at four different cancer types, including CRC, in rural Western Australia and found that surgery OOP costs were the highest [18]. Surprisingly, the total appointment OOP costs for all specialists were more expensive than the OOP costs of chemotherapy and radiotherapy individually. This may be because many specialists see patients in their private rooms as the public hospital outpatient clinics may not be available or be suitable. Private rooms are expensive to maintain, and so specialists need to charge to cover their costs. Furthermore, OOP costs are influenced by the fact that medical scheduled rebates have not kept up with inflation, with only an annual average indexation of 1.1% [19].

Interestingly, in a country with a universal healthcare system such as Malaysia, Azzani et al. showed that patients had some level of financial difficulty at the time of their diagnosis (94.2%), and at the 6-month (85.5%) and 12-month follow-up period (77.2%) [17]. To cope with the financial burden, most patients used their current income and savings, like the findings of the present study. Similarly, Veenstra et al. identified patients with CRC from the surveillance, epidemiology and end results cancer registry and found that most had some level of financial burden (62%) and had to cut down on expenses in general and use savings [12].

Studies from countries such as Ireland, which has a broadly similar mixed public–private healthcare system as Australia, show that the majority of patients with CRC have no financial stress (59.1%) or financial strain (60.7%) during their treatment [20]. However, and consistent with the present study findings, 20% of patients relied on other sources of funds to cover any OOP costs [20]. This suggests there is still more work to be done to improve financial support for patients. In the Australian setting, one option to address this is through the various state cancer councils. Finally, Gordon et al. used a state-based cancer registry to identify patients with CRC in Australia, and consistent with our findings, they showed that most patients were financially comfortable (> 70%) and were not experiencing financial strain (> 85%) both at 6 and 12 months after diagnosis. Many patients used their savings to pay for any OOP expenses [21]. Overall, this reflects well on the Australian healthcare system, which mostly provides treatment in a timely and effective manner with minimal OOP costs and a taxpayer funded safety net (Medicare) for uninsured patients. However, it may also because Australia is a developed country, and many in the community have the financial means to cover OOP expenses. It is not possible to determine from the data in the present study whether patients who paid higher OOP costs than others had received either more timely or more effective treatment. The example of the single patient who self-funded their medical oncology treatment ($57,500) highlights the fact that the PBS in Australia shields many patients from potential large OOP costs when undergoing cancer treatment.

Certainly, those who had children and were young were more likely to express financial burden or stress than older patients or those without children. Hanley et al. also found that patients with dependents expressed a greater likelihood of financial strain, consistent with our findings [20]. This is likely explained by the financial burden usually associated with supporting a growing family. This was also consistent with a study by Veenstra et al., where younger patients had a higher financial burden for treating CRC than older patients [12]. Similarly, Huang et al. in China found that younger patients who were employed were more likely to have a higher financial burden than retirees [22]. This may be due to the unpaid time taken off from work for treatment or the financial impact of developing complications following treatment. Brink et al. assessed the employment status of patients with different cancer types in Denmark. They showed that patients were less likely to return to work 1 and 3 years after a cancer diagnosis in comparison to non-cancer patients [23]. In Australia, loss of income as a result of a diagnosis of cancer is not just personal but also has an indirect negative impact on the wider community, with an estimated reduction of $1.7 billion annually in the gross domestic product as a consequence of lost productivity [24].

The limitations of the present study include only providing a single time point, long-term cross-sectional representation of the financial burden and stress of patients who undergo liver resection for CRLM rather than making assessments at different time points. The cohort studied consisted of patients who had liver resection with curative intent for CRLM, and while most patients were cured, 20% of patients who completed the survey had recurrent disease and were undergoing treatment at the time of responding. While it was not possible to ask deceased patients or their family members about the financial stress and worry and whether the costs and treatment were worthwhile, this study of a blended cohort of patients who were apparently cured and those with recurrent disease provides some valuable insights. It is worth noting that the study analyses began from the diagnosis of liver metastases, which means that many patients had already undergone colorectal surgery prior to the starting point. This is likely a key reason why the OOP costs associated with colorectal surgery were lower than those for liver surgery in this analysis. Furthermore, liver resections can be more complex than the primary bowel surgery, and occasionally, patients require multiple resections, which may explain the discrepancy in OOP costs between these two types of surgery.

The mapping of each patient’s cancer journey to determine the OOP costs was performed retrospectively, and for the sake of simplicity, it was assumed that all specialists charged the AMA rates. Furthermore, an assumption was also made that imaging, blood tests, and general practitioner visits would not result in OOP and were fully funded by Medicare. Also, this study did not examine the fees of the assisting surgeon and anaesthetist, and ultimately, this may result in some underestimates and approximations in the OOP costs. Regardless, the study’s findings demonstrate manageable costs and limited patient financial burden and stress. Additionally, the study cohort was treated in a relatively affluent part of Australia where private health insurance rates are greater than the national rate (66% vs. 44.8%). Therefore, the financial toxicity from OOP costs, financial worry, and stress may not necessarily be representative of all socioeconomic groups in Australia. Future studies should examine how private insurance coverage levels affect financial burden and access to care in other Australian states and compare public and private care pathways. Furthermore, due to the cross-sectional nature with a small sample size, causal inferences with financial burden and stress could not be assessed, although an association of these factors was evaluated. The final limitation is that this study consisted of patients who underwent liver resection by a single surgeon, and the cohort was relatively small due to the number of deceased patients and non-responders. Despite this, by international standards, it is still a large sample, and the significance of the study is that this is the first in patients with CRLM to assess the OOP costs, financial burden, and stress in a cohort of patients who have undergone liver resection with long-term follow-up.

## Conclusion

This study demonstrates a manageable amount of OOP costs in patients who undergo liver resection for CRLM. Most patients were not financially worried or stressed with the cost of their treatment. Understanding the costs and burden of disease can assist patients in better planning for the future and provide policymakers with areas to improve. As cancer care in Australia becomes more complex and expensive, this study may also be an important stimulus to begin discussions about the relative costs and value of specific treatments for different cancer types.

## Supplementary Information

Below is the link to the electronic supplementary material.

ESM 1 (DOCX 17.4 KB)

## Data Availability

De-identified data may be obtained by requesting the corresponding author.

## Code availability

Code may be obtained by requesting the corresponding author.
