# Supplementary material for: Out-of-pocket medical costs in relation to resection of colorectal liver metastases in the Australian healthcare system
Source: Support Care Cancer. 2025 Jun 24;33(7):621. doi: 10.1007/s00520-025-09669-2 (PMC12187899; doi:10.1007/s00520-025-09669-2)
Supplement: Supplementary file 1 — (DOCX 17.4 KB) [file 520_2025_9669_MOESM1_ESM.docx]

Appendix 1

Section 1: Demographic details

Could you please provide a few details about yourself? These questions aim to know how answers vary across respondents depending on these circumstances. All answers will be treated as confidential. Please circle the correct answer or indicate the correct number or description as necessary.

Question 1

Are you?

Male ...............................................................................1

Female............................................................................2

Question 2

What is your date of birth? Please indicate below.

Day ……………….. Month………….…..Year…………………

Question 3

What is your marital status? Please circle the appropriate answer (use ‘response’ if preferred).

Single..............................................................................1

Married or living as married.............................................2

Separated .......................................................................3

Divorced.........................................................................4

Widowed........................................................................5

Other (**SPECIFY**)...........................................................6

Question 4

What is your employment status? Please circle the appropriate answer.

In full-time work..............................................................1

In part-time work............................................................2

Currently seeking work....................................................3

Homemaker....................................................................4

Retired............................................................................5

Disabled ……………………………………………………………………….6

Other (**SPECIFY**)...........................................................7

Question 5

What is the highest level of education you completed? Please circle the appropriate answer.

Primary........................................................................................................1

Some secondary/high school.......................................................................2

All secondary/high school............................................................................3

Vocational Education Training e.g. TAFE/College ..........................................4

Undergraduate University ............................................................................5

Post-graduate university (SPECIFY) ..............................................................6

Question 6

Could you please provide an estimate of your annual household income from all sources (before tax and other deductions and including your partner/spouse)? Please circle the appropriate answer.

Less than $19,999………………………………………………………1

$20,000 to $39,999 ……………………………………………………2

$40,000 to $59,999 ……………………………………………………3

$60,000 to $79,999 …………………………………………………….4

$80,000 to $99,999 …………………………………………………….5

$100,000 to $119,999 ………………………………………………..6

$120,000 to $139,999 …………………………………………………7

$140,000 to $159,999 …………………………………………………8

$160,000 to $179,999 …………………………………………………9

$180,000 to $199,999 …………………………………………………10

Above $200,000 ………………………………………………………….11

Question 7

Who is the main earner in the household? Please circle the appropriate answer.

Cancer patient……………………………………………………..1

Spouse/Other………………………………………………………..2

Shared between survivor/spouse…………………………..3

Question 8

Do you have private health insurance? Please circle the appropriate answer.

Yes ………………………………………………………1

No ……………………………………………………….2

Question 9

How many adults are there in your household? Please indicate the number of adults below.

Number of adults_________

Question 10

How many children are there in your household? Please indicate the number of children below.

Number of children___________

## Section 2: Financial Burden and Worry

This section tries to understand the financial burden and worry that you had when trying to make payments for your diagnosis, treatment and follow-up of your **Colorectal Liver metastases**. This section is a general assessment of the financial burden you have felt since the diagnosis of your **Colorectal Liver metastases.**

Question 1 (Financial worry/stress)

How much do you worry about financial problems that have resulted from your Colorectal Liver metastases and its treatment?

Not at all ………………………………………………………………..1

Slightly ……………………………………………………………………2

Moderately/Somewhat ………………………………………….3

Very much……………………………………………………………….4

Extremely………………….…………………………………………….5

Question 2 (Financial burden)

How has the diagnosis of Colorectal Liver metastases impacted on the ability to make ends meet?

Very Difficult……………………………1

Difficult……………………………………2

Somewhat Difficult………………….3

Normal…………………………………….4

Somewhat Easy……………………….5

Easy…………………………………………6

Very Easy……………………………7

Question 3 (Financial burden)

My illness has had no impact on my finances

Strongly disagree………………………………….1

Disagree………………………………………………..2

Somewhat disagree……………………………….3

Neither agree nor disagree……………………4

Somewhat agree……………………………………5

Agree…………………………………………………….6

Strongly agree……………………………………….7

Question 4

We would like to learn about how the diagnosis, treatment and follow up of your Colorectal Liver metastases affected your finances. Please check ALL of the responses below that apply.

I had to use savings ………………………………………………………………………………………1

I had to borrow money or take out a loan …………………………………………………….2

I could not make payments on credit cards or other bills ……………………………..3

I cut down on spending for food and/or clothes ……………………………………………4

I cut down on spending for health care for other family members ……………….5

I cut down on recreational activities ………………………………………………………………6

I cut down on expenses in general …………………………………………………………………7

I had to sell some of my personal items/possessions e.g. car, jewellery ......…8

I had to sell stocks or investments………………………………………………………………….9

I had to sell my home …………………………………………………………………………………….10

Question 5

How would you describe your reaction to the cost of treating cancer?

Spent more money than I expected……………………1

Spent about what I expected……………………………...2

Spent less than I expected…………………………………..3

Question 6

How would you describe your reaction to how your medical benefits covered the cost of treating cancer?

Contributed more money than I expected…………………………..1

Contributed about what I expected………………………………………2

Contributed less money than I expected……………………………..3

Question 7

When choosing a treatment course for your cancer, did you consider the cost of treatment?

No, not at all………………………………………….1

Yes, a little bit……………………………………….2

Yes, a great deal…………………………………..3

Question 8

Did you decide not to have a recommended cancer treatment because it was too expensive?

No……………………………………………………………..1

Yes…………………………………………………………….2

Do not know/not sure………………………………3
